# Effects of larvae density and food concentration on Crown-of-Thorns seastar (*Acanthaster cf*. *solaris*) development in an automated flow-through system

**DOI:** 10.1038/s41598-017-19132-w

**Published:** 2018-01-12

**Authors:** S. Uthicke, M. Liddy, F. Patel, M. Logan, C. Johansson, M. Lamare

**Affiliations:** 1 Australian Institute of Marine Science, PMB No 3, Townsville, Queensland 4810 Australia; 2 Department of Marine Science, University of Otago, 9016 Dunedin, New Zealand

## Abstract

Coral-eating Crown-of-Thorns Sea stars (*Acanthaster* spp.) are major contributors to coral reef loss in the Indo-Pacific region. A release from food limitation of their planktotrophic larvae through enhanced pelagic productivity is one of the main hypothesis explaining population outbreaks (‘nutrient limitation hypothesis’). To improve the understanding of these outbreaks we developed an automated flow- through larvae rearing system that maintained food (microalgae) at set levels over the course of four 15d experiments. This resulted in stable food concentrations in experimental tanks. Increased algae concentrations had a significant positive effect on larval development and size at 10 and 15 days post fertilization (dpf). Larvae densities had no effect at 10 dpf. At 15 dpf greater larvae densities were associated with declines in larvae size. Larval development was slowed under higher larvae densities. Thus, the effects of algae concentration and larvae density were additive at 15 dpf, with larvae under low densities at a given algae concentration being further developed than those under higher densities. The development of a flow-through system gives greater insight into the effect of algae and larvae concentrations on *Acanthaster* development, and the system can be applied to further test the nutrient-limitation hypothesis for present and future outbreaks.

## Introduction

Several species of the corallivorous Crown-of-Thorns Seastar (CoTS) *Acanthaster* spp. (until recently grouped as *A*. *plancii*) are distributed across coral reefs of the Indo-Pacific Ocean^1,2^. Most scientific research has focused on the Pacific species currently referred to as *A*. *cf*. *solaris*^3^. All species exhibit episodic population explosions, or outbreaks^4^, as is observed in a range of echinoderm species with planktotrophic larvae^5^. These outbreaks can lead to substantial loss of coral cover, often on par with other disturbances such as bleaching or cyclones. This has been well-documented on Australia’s Great Barrier Reef (GBR), where up to 42% of coral loss was attributed to consumption by *Acanthaster cf*. *solaris*^6,7^. The GBR is presently experiencing its fourth outbreak since the 1960’s^4,8^, with the outbreak following previous patterns of starting in the Northern or Cairns section of the GBR and subsequently moving southwards^9^.

Despite intense studies since the early 1980’s, the exact causes of the outbreaks are not fully resolved or agreed upon amongst scientists. Overfishing of potential CoTS predators, either of Triton snail^10^ or finfish^11^ was amongst the early hypotheses explaining population outbreaks. More recent work has identified that, indeed, a variety of predators on all CoTS life-history stages exist, including those feeding on larvae stages^12,13^ while scars of sub-lethal predation can be found on about 30 to 60% of small and large CoTS, respectively^14^.

The “nutrient limitation hypothesis” is usually seen as an alternative to the overfishing hypothesis to explain either primary outbreaks^15^, the outbreak spread, or secondary outbreaks^4,16^. In essence, this hypothesis poses that growth and development of the planktotrophic *Acanthaster* spp. larvae are naturally limited by the concentration of their planktonic food (primarily phytoplankton > 2 µm). This limitation may be removed following periods of increased terrestrial runoff of inorganic nutrients into the coastal system, which promotes increased phytoplankton biomass^15,17,18^. Indeed, laboratory experiments conducted during the first two outbreaks on the GBR confirmed that CoTS larval development is accelerated by increased food supply, although in some cases, highest concentrations (>5,000–10,000 cells mL^−1^) can render development more variable^19^. A variety of further experiments during the third outbreak on the GBR and in Okinawa, Japan summarized in: refs^17,18,20^ and during the current outbreak on the GBR^21–24^ generally confirmed that larvae undergo faster development under higher food concentrations. Due to likely high mortality rates in the plankton seen in invertebrate pelagic larvae^25^ including echinoderms^26^, shorter developmental times will increase larvae survivorship to settlement^23^.

There is ongoing debate on the nutrient hypothesis. While it is accepted that larva grow faster under higher food concentrations, it is still unclear how limiting actual natural phytoplankton concentrations are for CoTS larval development in the field^8^. Given the limited amount of available data on phytoplankton species composition, quantity and nutritious quality, ‘threshold values’ for accelerated larvae growth or development are usually discussed in terms of chlorophyll concentrations as a proxy for micro algae biomass^17,18^. This pragmatic approach however has several intrinsic problems of interpretation. Firstly, chlorophyll values measured *in situ* or via remote sensing do not distinguish algae size and will include food items too small or two large to be consumed by CoTS larvae. Secondly, cell-specific chlorophyll content between natural algae and those used in experiments can differ by orders of magnitude, and organic or energy content vary distinctly between these algae^27^. For instance, a recent set of experiments employed an alga with extremely low cell-specific chlorophyll values (the flagellate *Proteomonas sulcata*) and demonstrated that larvae can complete development under very low chlorophyll *a*^21,22,24^. However, algae cell numbers required for this were high compared to the low cell densities found on the GBR in non-flood situations^28,29^.

Here, we addressed two additional issues associated with experimental investigations of the nutrient hypothesis and threshold phytoplankton concentrations for *Acanthaster* spp. larval development. Firstly, we recognized that all previous experiments were conducted in batch culture mode with non-continuous feeding to a nominal target cell number or chlorophyll concentration, with feeding occurring between 0.5 and 3 times per day (see Supplementary Table 1). This approach means that chlorophyll values used for threshold calculations represent peak values per treatment, and not a continuous or average supply. To overcome this, we developed a culture system that keeps larvae in flow-through conditions and under continuous algae food supply. Secondly, previous experiments on larvae were conducted with varying larvae densities, generally between 0.25 and 2 larvae ml^−1^ (see Supplementary Table 1). Recently developed genetic techniques^30–32^ measured the highest larvae densities during the spawning season in Okinawa and the GBR of around 50 larvae m^−3^ (=0.00005 ml^−1^). Thus, all inference on larval ecology available to date has been derived from experiments using larvae densities 4–5 orders of magnitude higher than naturally occurring.

Here, we present data from a set of four large-scale flow-through experiments that offered algae at concentrations (300 to 1000 cells ml^−1^) to larvae kept at three densities (0.5, 1 and 2 larvae ml^−1^). Given that work under natural larvae densities is practically unachievable, the intention of manipulating larvae densities was to test if density variation in the range commonly used in previous experiments has the potential to alter the inference drawn from these studies.

## Results

### Parameters in the four experiments

We conducted a total of four independent experiments, with the first three (in 2015) using only one algae concentration in the header tank, and the final experiment having two algae cell concentrations in separate header tanks. The initial experiment (2015-1) used an inflow rate of 50 ml minute^−1^ (~5 turnovers d^−1^) into each 14 L cone. At that flow rate, in tank algae concentrations were reduced by more than 50% through larvae feeding, with highest reductions under high larvae density (Table 1). Flow rates were tripled to 150 ml minute^−1^ in the subsequent experiments which narrowed the gap between inflowing algae concentrations and in tank values.

**Table 1:** Details for treatment and measured conditions for four experiments on *Acanthaster cf*. *solaris* development undertaken in 2015 and 2016. Treatments were high and low chlorophyll and three larval densities (0.5, 1 and 2 larvae mL^−1^). In 2015 the two chlorophyll conditions were tested separately in the experiments, high chlorophyll was tested in experiment 1 and 3 and low chlorophyll in experiment 2. *Larvae density* (larvae mL^−1^ ± SD) measurements were taken 5 times during all experiments (n = 5, exception of experiment 3 high chlorophyll n = 3. The *flow rate* (mL min^−1^ ± SD) was set manually during the experiment (experiment 1 and 2 n = 2, and experiment 3 n = 3). The chlorophyll concentration entering the cones was measured as *chlorophyll a inflow* (µg L^−1^ ± SD) (experiment 1 and 3 n = 2, and experiment 2 n = 3) and checked against *algae concentration inflow* (cells mL^−1^ ± SD) (experiment 1 n = 3, and experiment 2 and 3 n = 2). To determine algae consumption rates the *algae concentration in the cones* were sampled (cells mL^−1^ ± SD) (experiment 1 and 2 n = 3, and experiment 3 n = 2). In 2016 both the high and the low chlorophyll treatments was completed in the experiment. *Larvae density* (larvae mL^−1^ ± SD) measurements were taken 5 times during all experiments (n = 5), and the *flow rate* (mL min^−1^ ± SD) was set 5 times during the experiment (n = 5). Samples for *chlorophyll a inflow* (µg L^−1^ ± SD) (n = 9) were checked against *algae concentration inflow* (cells mL^−1^ ± SD) (n = 3). To determine algae consumption rates the *algae concentration cones* were sampled (cells mL^−1^ ± SD) (n = 3). Hypothetical cell specific chlorophyll values for the algae mix were calculated for all experiments in 2015 and 2016 based on parallel readings of chlorophyll and algae concentrations in the inflow water. *Steady state chlorophyll a cone* (µg L^−1^) of each larvae density was calculated based on the *algae concentration cones* and cell specific values (in 2016 averaged over both algae density treatments because these were derived on the same algae stocks).

Experiment	Target conditions		Measured conditions					
	Chlorophyll treatment	Larval density (mL^−1^)	Flow rate (mL min^−1^)	Chlorophyll *a* inflow (µg L^−1^)	Algae concentration inflow (cells mL^−1^)	Steady state chlorophyll *a* cone (µg L^−1^)	Algae concentration cone (cells mL^−1^)	Larval density (mL^−1^)
2015-1	0.8			0.65 (0.12)	1481 (65)			
		0.5	53.30 (1.6)			0.33	761.00 (28.31)	0.44 (0.08)
		1	53.00 (0.9)			0.22	505.83 (61.57)	0.77 (0.02)
		2	53.30 (9.8)			0.21	481.44 (76.41)	1.58 (0.21)
2015-2	0.4			0.43 (0.03)	725 (92)			
		0.5	144.60 (4.2)			0.33	557.67 (68.42)	0.50 (0.06)
		1	149.00 (1.4)			0.32	539.67 (83.86)	0.86 (0.17)
		2	146.40 (1.7)			0.25	428.00 (120.24)	1.78 (0.22)
2015-3	0.8			0.80 (0.07)	1139 (387)			
		0.5	150.00 (11.3)			0.72	1031.00 (186.76)	0.52 (0.07)
		1	147.40 (1.7)			0.72	1019.56 (181.39)	0.76 (0.10)
		2	149.40 (3.3)			0.63	892.44 (258.01)	1.84 (0.09)
2016-1	1.4			1.41 (0.38)	1479 (353)			
		0.5	140.00 (5.2)			1.01	1074.89 (269.56)	0.61 (0.07)
		1	149.20 (6.5)			0.87	932.33 (308.23)	1.11 (0.13)
		2	148.30 (7.9)			0.71	760.00 (83.16)	2.28 (0.13)
	0.6			0.60 (0.04)	640 (53)			
		0.5	151.12 (6.5)			0.45	480.67 (15.28)	0.62 (0.07)
		1	153.28 (4.9)			0.38	402.11 (19.68)	1.12 (0.13)
		2	150.50 (6.4)			0.28	302.44 (37.51)	2.22 (0.10)

Average larvae densities per treatment were close to nominal target values and well distinct among the treatments (Table 1). Overall, mortality rates after stocking the experiments were low (global average M = 0.03 d^−1^, SD = 0.02, details in Supplementary Table 2), with differences in mortality small among low density (average M = 0.04 d^−1^, 1 SD = 0.02), intermediate density (M = 0.03 d^−1^, 1 SD = 0.02), and high density (M = 0.02 d^−1^, 1 SD = 0.02).

### Larvae response

Development of larvae was scored at 10 and 15 dpf. In general, larvae developed well among treatments and only a small number of abnormal developing larvae were observed (details in Supplementary Figs 1 and 2). At 10 dpf, most larvae were late-stage bipinnariae or early-stage brachiolariae, with only larvae at higher algae concentrations reaching mid brachiolaria stage. Overall, 18% of larvae had reached the mid brachiolaria stage, and only 0.1% the late brachiolaria stage. Most larvae (global average: 90%) of all treatments reached the brachiolaria stage at 15 dpf, with 20% reaching late-brachiolaria stage. There was a clear trend towards more advanced development (i.e. to the mid- and late brachiolaria stage at higher food densities (Supplementary Figs 1 and 2).

**Figure 1:**
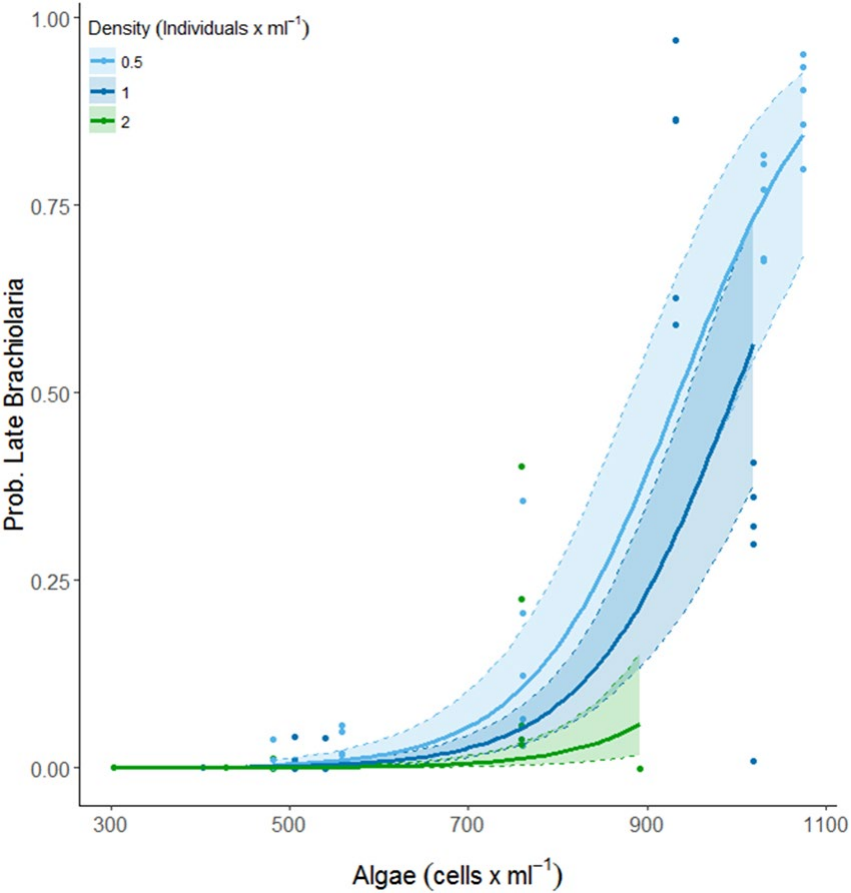
The modelled (see Table 2) proportion of larvae developed to mid-brachiolaria stage at 10 days post fertilisation. Confidence intervals derived from bootstrap (N = 1000) analysis, dots represent partial residuals at the tank level derived from the model.

We focused statistical analysis on mid brachiolaria stage at 10 dpf and late brachiolaria stage at 15 dpf. Models fitted for both 10 and 15 dpf suggested a strong positive dependence of the proportion of mid- and late-stage brachiolariae with food concentration, and there was support for an effect of algae density in both analyses (Table 2). At 10 dpf, neither larvae density nor the interaction between algae and larvae density explained a significant amount of the variation (Table 2). At algae concentrations of <700 cells ml^−1^, very few advanced larvae were observed (Fig. 1). Beyond that concentration, however, the proportion inclined steeply, with around 75% of all larvae being at mid-brachiolaria stage under higher food concentrations.

**Table 2:** Results of binomial linear mixed effects models testing the effect of algae cell concentrations (algae) and density of larvae (larval density) on the proportion of advanced stage (developed past early stage brachiolaria: >early brachiolaria) Brachiolaria larvae of *Acanthaster cf*. *solaris* 10 and 15 days post fertilisation. Models include an observation level random effect to account for over-dispersion. Note that intercept and slopes are for scaled data. Significance levels were tested by deviance tests (Χ^2^ and p values). The overall model explained 71% of the variance for mid-stage brachiolariaeat day 10 and 64% for late-stage brachiolariae at day 15, respectively.

		Estimate	SE	X^2^	p
Day 10					
Mid-stage brachiolariae	Intercept	−4.8238	0.4061		
	Algae	3.7874	0.3623	109.30	**<0**.**0001**
	Larval Density	0.0319	0.2865	0.01	0.9113
Day 15					
Late-stage brachiolariae	Intercept	−4.0709	0.3140		
	Algae	3.0524	0.2998	103.66	**<0**.**0001**
	Larval Density	−0.9393	0.2841	10.93	**0**.**0009**

**Figure 2:**
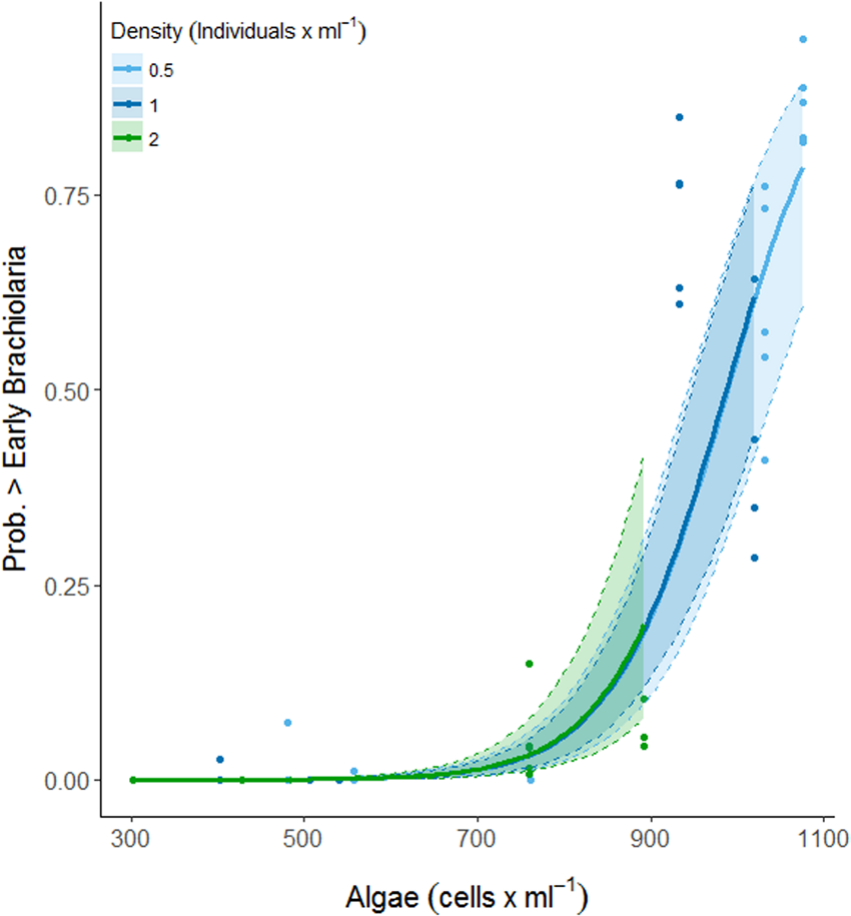
The modelled (see Table 2) proportion of larvae developed to late-stage Brachiolaria at 15 days post fertilisation. Confidence intervals derived from bootstrap (N = 1000) analysis, dots represent partial residuals at the tank level derived from the model.

**Figure 3:**
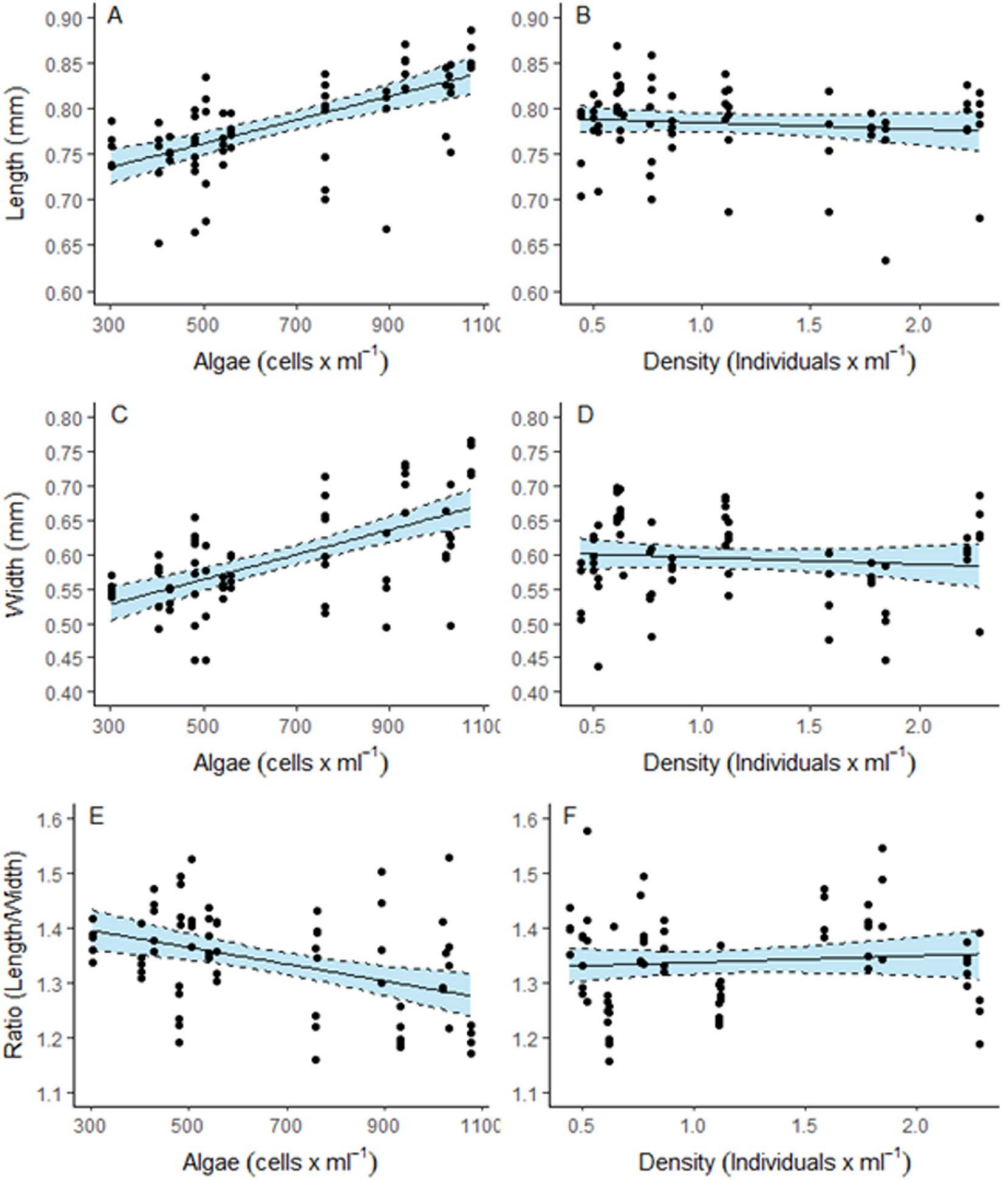
Modelled larvae length (**A**,**B**), width (**C**,**D**) and ratio (**E**,**F**) against algae concentration and larvae density *of Acanthaster cf*. *solaris* larvae 10 days post fertilisation. Confidence intervals derived from bootstrap (N = 1000) analyses, dots represent partial residuals at the tank level derived from the model.

The probability of being late-stage brachiolaria at 15 dpf was significantly affected by algae concentrations and larvae density, with no significant interactive effect (Table 2). Under all three larvae densities, increasing food concentrations increased the probability of reaching the late-brachiolaria stage (Fig. 2). The probability of reaching a late-brachiolaria stage was negatively related to larvae density, with a small proportion of larvae reaching that stage at the highest food concentration achieved in the 2 larvae ml^−1^ cultures (Fig. 3).

### Larvae Size

Length and width of the larvae were determined at 10 and 15 dpf. At 10 dpf, larvae length and width increased significantly with algae cell concentrations (Table 3, Fig. 3), but were independent of larvae density. The ratio of length to width of the larvae at 10 dpf decreased significantly with increasing food availability, and was independent of larvae density (Table 3, Fig. 3). Thus, well-fed larvae were wider in relation to their length.

**Table 3:** Results of linear mixed effects models testing the effect of algae cell concentrations (Algae) and density of larvae (larval density) on the length, width and length to width ratio (ratio) of *Acanthaster cf*. *solaris* larvae 10 and 15 days post fertilisation. Significance levels were tested by deviance tests (*Χ*^2^ and p values). Interactions with p > 0.25 in initial model fits were removed from final models. Tank was included as random factor in the models and the percentage variance (%) explained by this factor given. The variance explained by the total model was 40 (length), 53 (width) and 33% (ratio) at day 10; and 62 (length), 67 (width) and 45% (ratio) at day 15.

			Estimate	SE	Χ^2^	p
Day 10						
Length						
	Fixed	Intercept	0.70490	0.02007		
		Algae	0.00013	0.00002	37.57	**<0**.**0001**
		Larval density	−0.00723	0.00846	0.73	0.3926
	Random	Tank	27.8			
Width						
	Fixed	Intercept	0.48440	0.02875		
		Algae	0.00018	0.00003	35.65	**<0**.**0001**
		Larval density	−0.00981	0.01209	0.66	0.4172
	Random	Tank	40.0			
Ratio						
	Fixed	Intercept	1.42800	0.04151		
		Algae	−0.00015	0.00004	2.37	**0**.**0004**
		Larval density	0.01218	0.01749	0.48	0.4864
	Random	Tank	28.4			
Day 15						
Length						
	Fixed	Intercept	0.73840	0.04611		
		Algae	0.00020	0.00006	14.70	**0**.**0001**
		Larval density	−0.03418	0.03317	46.75	**<0**.**0001**
		Algae x density	−0.00008	0.00005	2.44	0.1180
	Random:	Tank	40.7			
Width						
	Fixed	Intercept	0.46610	0.06697		
		Algae	0.00028	0.00009	14.57	**0**.**0001**
		Larval density	−0.00855	0.04819	17.28	**<0**.**0001**
		Algae x density	−0.00010	0.00007	2.05	0.1521
	Random	Tank	54.59			
Ratio						
	Fixed	Intercept	1.49200	0.06418		
		Algae	−0.00017	0.00007	6.79	**0**.**0092**
		Larval density	0.02504	0.02701	0.8597	0.3538
	Random	Tank	41.7			

**Figure 4:**
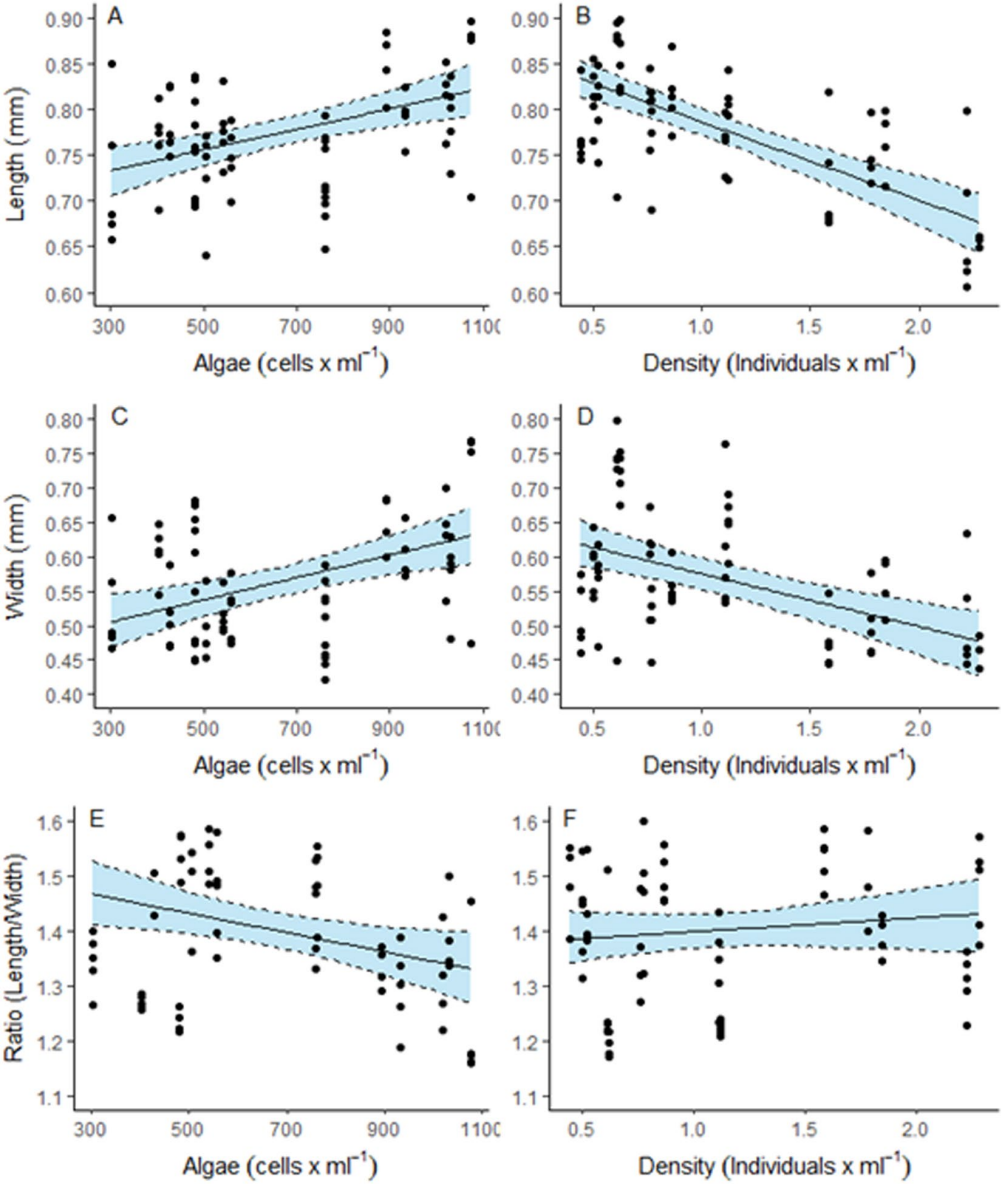
Modelled larvae length (**A**,**B**), width (**C**,**D**) and ratio (**E**,**F**) against algae concentration and larvae density *of Acanthaster cf*. *solaris* larvae 15 days post fertilisation. Confidence intervals derived from bootstrap (N = 1000) analyses, dots represent partial residuals at the tank level derived from the model.

At 15 dpf, food also had a significant positive effect on size and width relationships of larvae (Table 3, Fig. 4). At 15 dpf, however, larvae exhibit a significant negative relationship larval size with larvae density (Table 3, Fig. 4). The length to width ratio at 15 dpf was also significantly affected by algae concentrations, with ‘thinner’ larvae at lower food concentrations (Table 3, Fig. 4).

### Settlement

**Figure 5:**
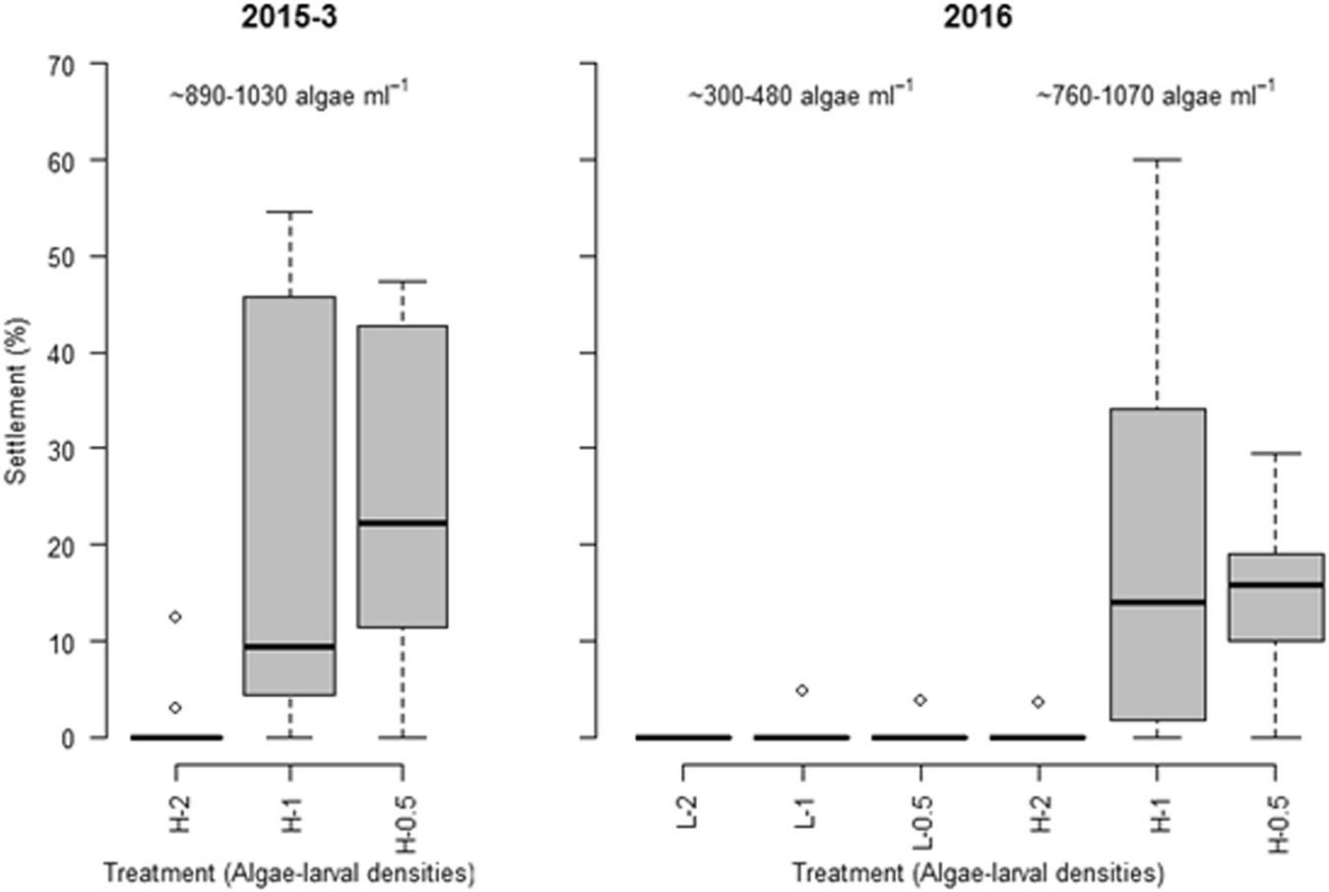
The percentage of larvae settling in different treatment conditions at the end of experiment 3 in 2015 and 2016. Well plates were used for settlement (n = 12 wells, except for the high chlorophyll treatment in experiment 3 in 2015 where n = 11).

Larvae settlement experiments were conducted at the end of experiments 2015–3 and 2016. In 2015, few larvae (average 1.4%) settled in the highest larvae density treatment (890 algae cells ml^−1^), whereas 20.6% and 25.6% settled in the 1.5 and 0.5 larvae ml^−1^ treatments, respectively (Fig. 5, 1020–1030 algae ml^−1^). In 2016, settlement rates were very low (0–0.4%, Fig. 5) in all nominal low algae (actual concentrations: ~300–480 cells ml^−1^) treatments. Similarly, only 0.3% of the larvae settled at the highest larvae density treatment, which had an algae concentration of 760 cells ml^−1^. The median and low larvae density treatments (algae concentration: 930 and 1070 cells ml^−1^) of the high algae treatment had distinctly higher settlement rates (20.0 and 14.5%, respectively).

## Discussion

In order to render experiments on larvae of the coral eating sea star *Acanthaster* spp. more realistic, we developed a continuous flow-through system to test whether developmental changes observed with increasing algae biomass were dependent on experimental larvae density.

The experimental system developed facilitated larvae culture under flow-through conditions at steady state algae concentrations. However, even at the higher flow rate used in the final three experiments, algae density displayed differences, albeit small, among larvae densities. Given CoTS larvae are more efficient at removing algae at higher cell densities^19,27^, algae concentrations in the tanks will achieve a steady-state equilibrium between food supply and consumption. However, given that the employed statistical design used the actual measured algae concentration and larvae densities for each treatment, this does not affect our interpretation of the results, because algae and larvae density effects were modeled separately, after partialling out the influence of larvae density and algae, respectively.

Larvae instantaneous mortality rates presented here are low (M ~0.03 day^−1^) compared to *in situ* rates estimated for echinoderms in general 0.06–0.26,^25^. Values in our flow-through experiments were also less than half of the mortality reported in previous batch experiments in the same facility^23^, and in a similar range as previously reported in laboratory experiments^22,24^. Poorbagher *et al*.^33^ observed larvae mortality rates in the New Zealand sea star *Sclasterias mollis* reared in the laboratory ranged from M = 0.15 to 0.02 d^−1^, with the rate of mortality generally decreasing with time over a 90 day period. Importantly however, in the latter study there was no significant effect of larvae nutritional status on mortality rate over the period.

Mortality rates reported here were measured in a predator free environment and are unlikely to reflect field values. In addition, rates presented here and by others are for the period after stocking the experiment, and do not include mortality of initial embryo and early larval stages, where losses can be high (ie. >50%, pers. observations). While little is known about predation upon *Acanthaster* spp. larvae in the field, planktivores such as damselfish, crabs and corals are known to eat larvae stages^12,13^ that will certainly cause higher *in situ* mortality. Other echinoderm larvae have also been observed to be eaten by common predators^25,34^, although rates of predation may be size-specific^35,36^, suggesting that predation on larvae may be complex and related to larval size, development rate, morphology and behaviour^34^.

Although not statistically tested, we observed a counter intuitive trend towards higher mortality rates in lower density treatments. We propose that this is likely to be an experimental artifact, with potential small losses of larvae during cleaning procedures having a proportionally higher impact at low densities.

As in all previous laboratory experiments e.g.,^17–19,23^, the amount of algae available to the larvae had a clear effect on size and speed of development in the larvae. Within the range of cell densities in our experiments (~300 to 1100 algae cells ml^−1^ steady state concentration, equivalent to 0.2–1.0 µg chlorophyll L^−1^), larvae size and development increased with algae concentrations. We did not observe the negative effect documented in some studies^21,22,24^ at extreme algae cell densities outside the natural range, including flood event conditions^28,37^.

At 10 dpf, larvae densities did not affect the larvae development and size. Development rate at all larvae densities steeply increases in algae cell concentrations >750 cells ml^−1^ (here 0.7 µg chlorophyll L^−1^), whereas length and width appear to increase continuously. Contrastingly, at 15 dpf, the results are more complex as larvae density exhibited an additive effect on larvae size and development.

At 15 dpf, probabilities to reach the late larvae stage increased with food availability for all densities, yet at lower densities larvae were more advanced at a given food concentration. Probabilities for being at late brachiolaria stage at the maximum algae densities achieved for the 2 ml^−1^ larvae density treatment were only 5.8% (95% Confidence Interval = 1.9–15.1%) at the high larvae density treatment. By contrast, these probabilities were 37.9% (23.6–54.7%) and 22.3 (13.7–33.3%) for 0.5 and 1.0 ml^−1^ larvae density treatments, respectively. At 15 dpf algae concentration had a significant positive effect on size. However, at 15 dpf higher larvae densities significantly reduced larvae size. Several previous studies documented increased *Acanthaster* larvae size with increasing food supply^18,21^, and similar responses have been seen in other echinoderms^38^, including in a number of sea stars.

A positive effect of food availability on sea star larval development has been observed for *Luidia clathrata*^39^, *Pisaster ochraceus*^40^ and *Asterina miniata*^41^. The latter author found size of *A*. *miniata* larvae significantly increased with food ration between 5 × 10^2^ and 5 × 10^5^ concentrations, while in the temperate sea star *Sclasterias mollis*, Poorbagher *et al*.^33^ observed diverging larvae size with time in larvae feed a high concentration algae diet (2000 cells ml^−1^ vs 8000 cells ml^−1^).

Food supply also affected larval morphology. Better-fed larvae were generally larger (i.e. wider) than those under low food concentrations as evidenced by the significant reduction in length to weight ratios with higher food. Morphological changes in echinoderm larvae across food concentrations are commonly observed^42^, which has been associated with changes in shape to optimize feeding at ambient food concentrations^43^. Food-induced changes in morphology have been observed previously in sea stars. Poorbagher *et al*.^33^ observed shape changes in the larvae of *Sclerasterias mollis*, specifically larvae feed high food levels (8,000 cells mL^−1^) being wider relative to length, compared with low food larvae. For *Acanthaster cf*. *solaris*, longer ciliated bands relative to total larvae size has been observed in food limited treatments^44^.

Finding morphological responses with changes in larvae nutrition might allow nutritional assessment of field caught larvae. Fennaux *et al*.^42^ and Lamare and Barker^26^ observed differences in larvae morphology between laboratory reared and field caught echinoid larvae, and concluded that these differences reflect food-limited larvae in the field. During field studies on *Echinometra*, where larvae were out-planted in a range of locations that varied in chlorophyll *a* concentrations, Lamare *et al*.^45^ observed no increase of larvae size with increased chlorophyll concentration, but larvae became more asymmetrical. Similarly, Poorbagher *et al*.^33^ noted differences in shape in larvae spawned from shallow versus deep-water populations of the sea star *Sclasteria mollis*, and suggested this reflects the nutritional status of the larvae due to differences in maternal investment in eggs. These observations suggest any differences seen in morphology could be used as a marker of spatial and temporal changes in *Acanthaster* larvae nutrition.

Settlement was only measured at the end of two of the four experiments, which means that we cannot uncouple the effects of food limitation and larvae density with the same rigor as for the other responses. Settlement rates observed are comparable to those found in other studies on *A*. *cf*. *solaris*, and are generally dependent on environmental factors such as larvae food supply and culture temperature^23,24^, coralline algae type^46^ or pre-conditioning on the coralline algae^47^. It is apparent that settlement reflects our assessment and analysis of the larvae stages: treatments with abundant late brachiolaria stage larvae also exhibited high settlement rates. For example, settlement after 15 dpf only occurred in appreciable numbers at higher algae cell concentrations (~>700 cells ml^−1^, in our experiments equivalent to 0.7 µg chlorophyll L^−1^).

The fact that larvae in some treatments did not settle after day 15 does not necessarily indicate that these would not complete development and settle at a later stage. Depending on food supply, Pratchett *et al*.^24^ observed settlement between 17 and 43 dpf, with peak values at 22 dpf. However, we argued previously that extended larval development duration and resulting higher exposure of larvae to predation and other mortality might be the ultimate mechanism how changes in water quality may influence CoTS population outcome^23^. As seen in the present study, larvae diet ration in the laboratory did not affect mortality rates in sea stars^33^, although at low food concentration larval development can be arrested^41^. We did, however, observe a positive relationship between larvae densities and reduced size as well as slower development, which may reflect competition for food within the cultures, or density-dependent negative culturing artifacts.

Survival of cultured echinoderms can be density-dependent within the range applied in the present study. For example, survival in *Lytechinus variegatus* larvae decreased from 76 to 68.5% between densities of 0.25 to 1.0 larvae mL^−1^ ^48^, especially during the middle stages of development. Reduced survival in higher density cultures of echinoids was attributed to mechanical abrasion between densely cultured larvae^48^. Sea stars have less rigid bodies and such mechanical damage may be less problematic. Nevertheless, it is possible that greater interactions in denser cultures of sea stars may interfere with feeding, while denser cultures are also more likely to suffer from poorer water quality and greater infection probability (see Bruno-Gomez-Gil 2000). Whether or not during CoTS outbreaks there is density-dependent mortality in wild larvae populations, either through competition (inter- and intraspecific) for food, pathogens or density-dependent predation, is unknown. Given that densities appear to be naturally much lower in *Acanthaster* spp. larvae populations (i.e. 0.00005 larvae mL^−1^), and that mechanisms of larvae mortality may not be comparable (i.e. predation in the field versus suboptimal conditions in the laboratory), the extrapolation of laboratory observations of survival to wild populations is difficult.

In summary, we demonstrated that *Acanthaster cf*. *solaris* larvae can be cultured successfully under flow-through conditions, thereby allowing future experiments under more realistic and more precisely defined nutritional conditions than is possible in batch culture. We propose that feeding experiments using ‘natural’ plankton under these conditions in combination with actual field measurements of algae composition, nutritious quality and densities will allow more realistic assessments of the ‘nutrient hypothesis’. Future experiments will also need to take into consideration that in natural plankton communities CoTS larvae will be in competition with other organisms grazing on the same food source. In addition, we illustrated that larvae densities can influence the experimental outcome and potential derivation of nutritional thresholds for enhanced growth. Although experiments under natural larvae densities, several orders of magnitude below those used here^30,32^, cannot practically be conducted, we recommend future experiments at or below 0.5 larvae ml^−1^. It appears that under lower larvae densities there is a ‘left shift’ of thresholds towards lower algae cell (or chlorophyll) concentrations. We hypothesize that an additional ‘left shift’ will be seen under continuous food supply. Thus, it is possible that enhanced growth can be triggered by algae cell concentrations somewhat lower then currently assumed. This can now be tested in targeted experiments. Furthermore, studies using genetic techniques to study small and large-scale temporal changes in CoTS larvae in the field are possible^30–32^. These allow correlation between larvae abundance and food quantity and quality, which will provide new opportunities to investigate the importance of nutrition in larval development and outbreak dynamics.

## Methods

### Experimental setup and feeding

Cone shaped 16 L (working volume: 14 L) Perspex vessels (“cones”) were used as experimental units (see pictures of experimental setup in the supplementary material). These were fitted with a Perspex lid with a hole for an aeration tube and banjo filter (Diameter: 90 mm) with a 206 µm mesh fitted at the outflow. Cones were supported in custom-made benches. In total, 15 cones were used for the experiments in 2015 and 30 for the experiment in 2016. The experimental room was temperature controlled at 28 ± 0.5 °C, and water temperature also set to 28 °C. Seawater was 0.04 µm filtered, and refiltered through an additional 1.0 µm bag filtration unit.

Pure algae strains were obtained from the Australian Algae Culture Collection (Hobart, Australia) and cultured at the Australian Institute of Marine Science. Algae were grown at 24 °C in a 12:12 light dark cycle in F/2 medium. Larvae were fed a mix of two algae species, *Dunaliella sp*. (CS-353) and *Tisochrysis lutea* (CS-177) previously used in larvae rearing experiments for *Acanthaster cf*. *solaris* larvae e.g.,^23,27^. Algae concentrations were fed in equal proportions in regards to cell-specific chlorophyll *a* content measured at the start of the experiments. This equated to cell count proportions of approximately 60% *Dunaliella sp*. and 40% *Tisochrysis lutea*.

In the 2015 experiments we supplied algae concentrations in a range presumed limiting to satiating, based on previous experiments with similar algae^19,20,23^. In 2016 we ran a low and a high algae concentration in parallel (using two header tanks and control units). In addition to food concentration, larvae density was used as a second experimental factor (see below).

### Design of flow-through system

Larvae were fed via an automated continuous flow-through system. The system used an interface panel (Siemens) that allowed setting respective chlorophyll *a* treatment concentrations at the start of each experiment. On a daily basis, algae stock concentrations were determined using flow cytometry and algae requirements were then calculated for a 24 hour period. An elevated 70 L feeder tank was dosed with the calculated algae stock that was gravity fed to the 500 L header tank. The delivery of the respective chlorophyll *a* concentrations was controlled by a feedback loop system, consisting of fluorescence sensors (WET labs, FLS and FLNTU), housed in each treatment tank monitoring chlorophyll *a* concentrations and controlling solenoid valves (Burkert) injecting concentrated algae stocks from the feeder tank. Software was set so that solenoid valves opened once chlorophyll values were below target values, and closed again once values were above those. This enabled the continuous and controlled delivery of chlorophyll *a* treatment concentrations. The system logged chlorophyll *a* concentrations every 10 sec. The treatment tanks delivered water using magnetic pumps (Iwaki) to the 14 L cone, where final flow rates were set for each cone. The fluorescence sensors were calibrated according to the manufactures protocol. As an additional check, at the beginning of the experiments a calibration test was carried out on the fluorescence sensors, by manually dosing the treatment tanks using the experimental algae cell concentrations in 0.2 µg L^−1^ chlorophyll *a* increments to a final value of 1 ugL^−1^, to cover the chlorophyll *a* treatment range. Concurrent samples were collected at each incremental step for chlorophyll *a* and cell density estimates. Sensor output readings were then compared with laboratory determined chlorophyll *a* concentrations (cell counts used as additional check). A calibration offset was applied, as required.

### Description of Experiments

*Acanthaster cf*. *solaris* specimens were collected in early November 2015 and 2016 at reefs between the Cairns and Port Douglas (16.5–17.0° S) in the Cairns Section of the GBR. Specimens were transported to the National Sea Simulator facility at the Australian Institute of Marine Science, Townsville, and kept in 1000 L flow-through tanks under ambient water temperature until used for experiments.

A minimum of 6 males and 6 females were spawned for each experiment using 1-methyladenine as described in^23,49^. As per the latter reference, young (pre-feeding) larvae were kept in four, 70 L fiberglass cones for the first two days post fertilization (dpf), with daily washes and removal of unfertilized oocytes and dead larvae from the bottom of the cones.

Experiments were stocked at 3 dpf. Prior to this, all larvae were subjected to a final wash over a 20 µm mesh and pooled. Density of the pooled stock was counted in 6 replicates under a stereo microscope and volumes required to add into each individual 14 L cone calculated to achieve nominal densities of 0.5, 1.0 and 2 larvae ml^−1^. Actual densities in each cone were counted subsequently (see below) and in a few cases densities corrected to the required densities.

### Larvae density, development (day 10 and 15) and settlement

Larvae densities were determined five times throughout each experiment after stocking the replicate larvae cones. To achieve this, cones were carefully mixed with a custom made plunger, while removing 6 subsamples for each replicate using a 5 ml syringe. The volume withdrawn was adjusted (3 to 8 ml) for each nominal density to allow sufficient larvae to be counted in each subsample. The number of larvae in each sub-replicate was counted directly after collection under a stereo microscope.

Developmental stages of the larvae were scored at 10 and 15 dpf. Larvae from each replicate were collected during the cone washing process, when the larvae were concentrated on the 20 µm mesh. Scoring for each experiment was conducted ‘blind’ by the same person (SU), that is, without knowledge of treatment. At least 100 larvae for each replicate were classified under 50×-magnification as either abnormally developed larvae, bipinnariae, early-, mid- or late-stage brachiolariae following Uthicke *et al*.^23^.

Subsequent to scoring, larvae were relaxed in 6.8% MgSO_4_, fixed in 4% Paraformaldehyde and photographed as described in Uthicke *et al*. (2015). Total larvae length and width were measured from a minimum of 20 larvae for each replicate as in Lamare *et al*.^49^.

Larvae settlement experiments were conducted after final scoring at 15 dpf in experiments 2015-3 and 2016. Larvae from the five replicates per treatment combination were pooled, and settlement experiments conducted in two 6-well plates (N = 12) for each treatment. We used small chips of the crustose red algae *Lithothamnium pseudosorum* as a known settlement inducer^46^ and scored settlement after 44–48 h of exposing the larvae to the substrate^47^.

### Determination of cell counts and chlorophyll *a* levels in algae stock cultures

Cell counts of the algae stock cultures were performed daily using Accuri C6 flow cytometer after samples were diluted 1/500 (Settings: 100 µl sample volume, fast fluidics speed, threshold: 80,000, detectors: FL1, FL3). Additionally, cell-specific chlorophyll *a* of the algae stock cultures was determined fluorometrically at the start of the experiment (Turner design 10 AU fluorometer). 100 µl samples were filtered through 0.45 µm GFC filters, ground to a slurry in 90% acetone and incubated in the dark for 2 hrs at 4°C (Parsons *et al*. 1984).

To confirm target levels of the amount of algae provided to the system and each cone of larvae, duplicate cell counts and chlorophyll *a* samples were taken from each chlorophyll *a* treatments on three occasions during each experiment. 100 ml samples were analysed using methods described above. Furthermore, at several occasions samples were taken from all cones during each experiments. The samples were run undiluted, immediately after collection to negate the need to fix samples as previous tests with fixed samples gave unreliable results.

To document potential variation amongst cones, temperature (Sper Scientific hand meter) was routinely measured and flow rates determined manually and if required, adjusted during the experiments.

### Statistical methods

We estimated a daily mortality rate (M) from the decrease in larvae density during the period after stocking each experiment, based on average initial (N_0_ at day zero) and subsequent densities. Rates were estimated by the slope of a regression of ln (N_0_/N_t_) on t, with t being the time after stocking in days, and N_t_ the density (larvae ml^−1^) at time t.

We used generalized linear mixed effects models^50^ to explore the effects of algae concentration (food supply) and larvae densities on the developmental stages and larvae size at 10 and 15 dpf, separately. These analyses were conducted across all four experiments, with experiment and treatment averages of tank algae concentrations and average measured larvae densities as explanatory variables.

For the analysis of the developmental stages, we restricted statistical tests separately for 10 and 15 dpf to scores of the most advanced larvae present in significant numbers in the global data set. These were the mid-stage brachiolariae at 10 dpf (~18% of the global dataset at that time) and the late-stage brachiolariae at 15 dpf (20%). Counts of larvae at the respective stages and remaining larvae were used in a generalized linear mixed effects model with binomial error distribution. To allow comparisons of relative effect size of the explanatory variables, larvae and algae density was scaled (mean = 0, sd = 1) for statistical analysis. For ease of interpretation, effects plots and partial residuals were presented on back-transformed scales. Initial analysis showed that data were over dispersed (variance ≫ mean). To accommodate overdispersion, we added an observation level random effect into the final model. Interactions that lacked inferential support (p > 0.25) were removed.

The analyses of the size data (length, weight) were conducted using a model with a Gaussian error distribution. These analyses included “Tank” as a random variable. The amount of overall variance explained by generalized effects models was calculated using pseudo R^2^ ^51^ as implemented in the MuMln library^52^.

Confidence intervals for partial effects represented in trend plots were generated by bootstrapping the models 1000 times, each time with a random draw (sampling with replaced) of observed data. Larvae settlement data were only available from a subset of the experiments and results were unambiguous. Hence, these were not statistically analyzed, but were interpreted from boxplots. All statistical analysis were conducted in the R package^53^.

### Data availability

Most data are presented in the supplement or main text, all data are available from the authors.

### Ethical statement

Work with Echinoderms does not require ethical approval.

## Electronic supplementary material

Supplementary information
