# Author Correction: Effects of larvae density and food concentration on Crown-of-Thorns seastar (*Acanthaster cf*. *solaris*) development in an automated flow-through system

**DOI:** 10.1038/s41598-018-27937-6

**Published:** 2018-06-21

**Authors:** S. Uthicke, M. Liddy, F. Patel, M. Logan, C. Johansson, M. Lamare

**Affiliations:** 1 Australian Institute of Marine Science, PMB No 3, Townsville, Queensland 4810 Australia; 2 Department of Marine Science, University of Otago, 9016 Dunedin, New Zealand

Correction to: *Scientific Reports* 10.1038/s41598-017-19132-w, published online 12 January 2018

This Article contains an error in Figure 1, where the modelled proportion for late-stage Brachiolaria is shown instead of the modelled proportion for mid-stage Brachiolaria. The correct Figure 1 appears below.

**Figure 1:**
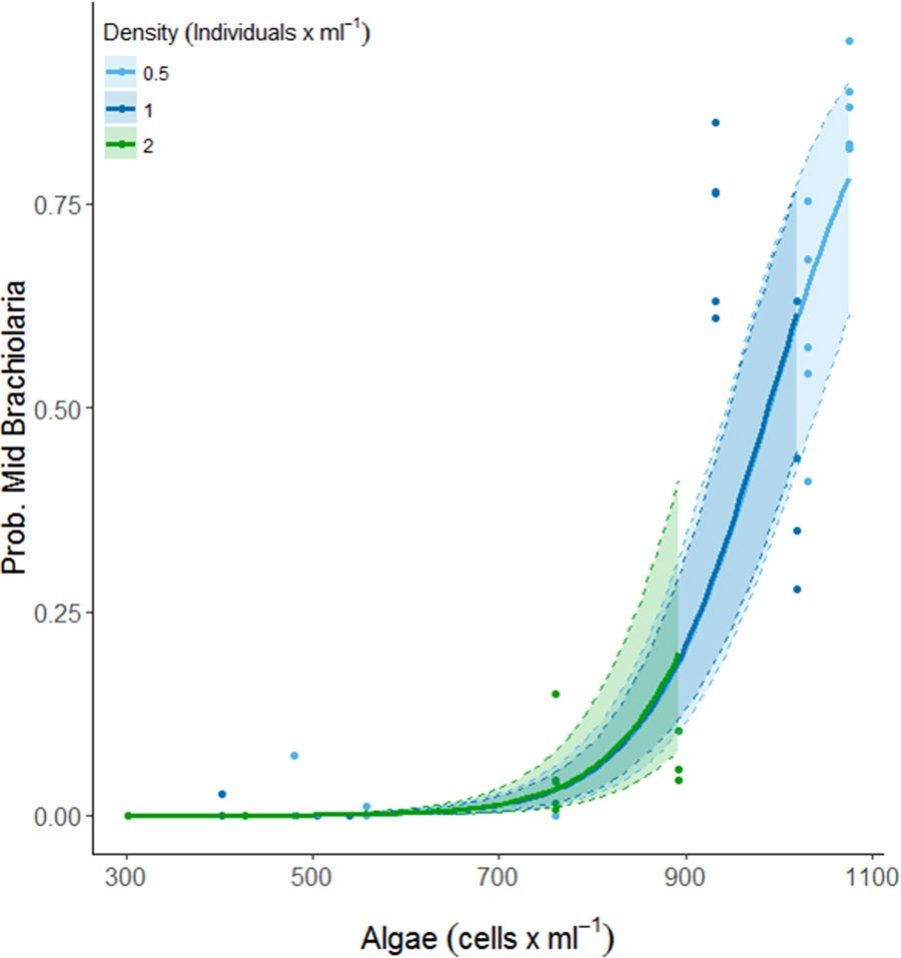
The modelled (see Table 2) proportion of larvae developed to mid-brachiolaria stage at 10 days post fertilisation. Confidence intervals derived from bootstrap (N = 1000) analysis, dots represent partial residuals at the tank level derived from the model.

In addition, this Article contains an error in Figure 2, where the modelled proportion for early-stage Brachiolaria is shown instead of the modelled proportion for late-stage Brachiolaria. The correct Figure 2 appears below.

**Figure 2:**
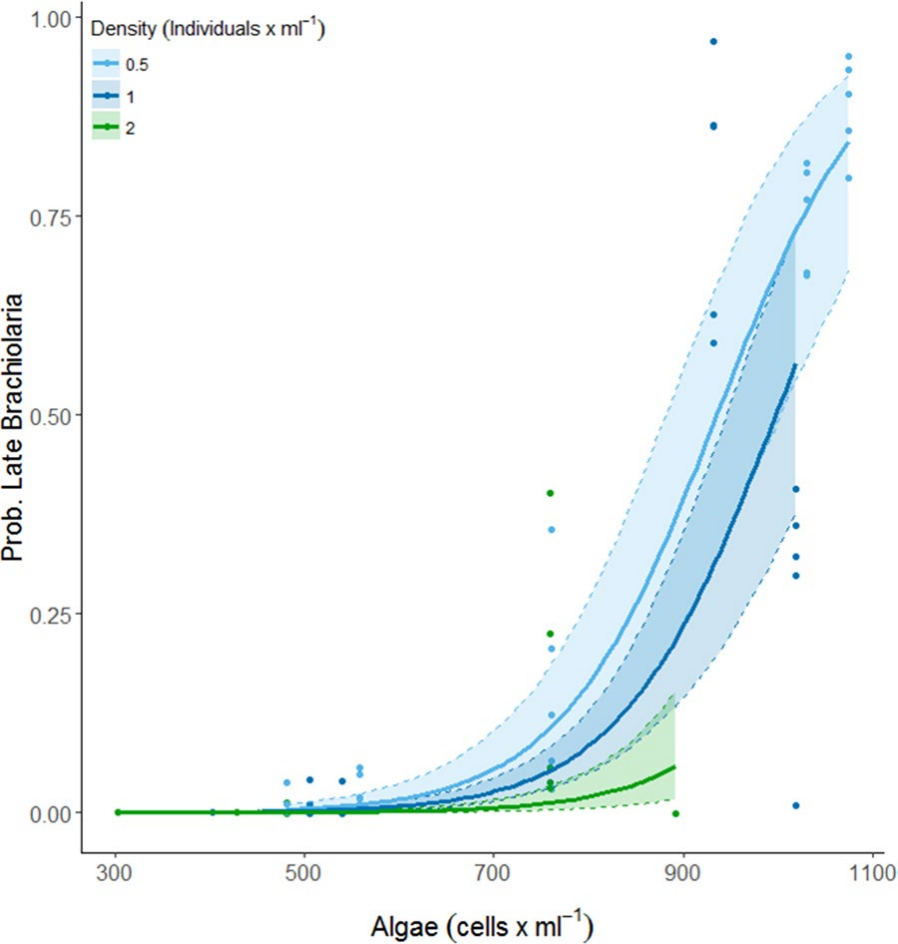
The modelled (see Table 2) proportion of larvae developed to late-stage Brachiolaria at 15 days post fertilisation. Confidence intervals derived from bootstrap (N = 1000) analysis, dots represent partial residuals at the tank level derived from the model.

