# Supplementary material for: Effects of larvae density and food concentration on Crown-of-Thorns seastar (Acanthaster cf. solaris) development in an automated flow-through system
Source: Sci Rep. 2018 Jan 12;8:642. doi: 10.1038/s41598-017-19132-w (PMC5766623; doi:10.1038/s41598-017-19132-w)
Supplement: Supplementary file 1 — Supplementary information [file 41598_2017_19132_MOESM1_ESM.pdf]

Effects of larvae density and food concentration on Crown-of-Thorns seastar (*Acanthaster cf. solaris*) development in an automated flow-through system

S. Uthicke, M. Liddy, F. Patel, M. Logan, C. Johansson, M. Lamare

Supplementary Table 1. Details of previous feeding experiments with *Acanthaster spp.* larvae. Eperimental locations suggest that all of these were conducted with larvae of *A. cf. solaris.* Ng: data not given in original reference.

**Reference**	**Purpose**	**Experimental treatments**	**Algae fed**	**Algae concentrations (cells mL^{-1})**	**Chlorophyll (µg L^{-1})**	**Type of feeding**	**Frequency**	**Larval Density (larvae ml^{-1})**
[1]	Temperature and salinity range, food quality	Temperature, salinity and different algae	*Dunaliella tertiolecta, Monocrysis lutheri, Amphidinium carteri*	3000	ng	Pulse	1 x per day	0.33
[2]	Test food limitation, clearance rates	Different algal concentrations, 2 types of algae	*Dunaliella tertiolecta, Phaeodactylum tricornutum*	0-50000	0-6	Pulse	0.5 x per day	0.25[1]
[3]	Study algae consumed	Different algae and ultraplankton	*Phaeodactylum tricornutum, Dunaliella tertiolecta, Tetraselmis sp. (and smaller plankton, all heat killed)*	550-7500	ng	Pulse	Once off	0.6
[4]	Test food limitation	Different algal concentrations	*Dunaliella tertiolecta*	3000-4000 (in Japan)			Not given	0.05-0.1
[5]	Selective feeding, study which organisms consumed	Different algae, cyanobacteria and micro beads	*Dunaliella tertiolecta, cyanobacteria*	500-5000 (for Dun.)	ng	Pulse	Once off	ng
[6]	Development and settlement assays	Ocean acidification	*Proteomonas sulcata*	33000-1000000	ng	Pulse	3 x per day	ng
[7]	Development and size	Temperature and ocean acidification	*Proteomonas sulcata*	25000–37000	ng	Pulse	Increasing with time from 1 to 3 x per day	Start with 12.5 eggs mL^{-1}

8	Development and size	Temperature and food density	*Phaeodactylum tricornutum, Dunaliella sp. and Chaetoceros sp.*	1100-9800	0.7-7.1	Pulse	2 x per day	1
9	Mortality, development and settlement	Algae concentrations	*Proteomonas sulcata*	0-100000	0-10	Pulse	1 x per day	1
10	Larval morphology	Algae concentrations	*Proteomonas sulcata*	0-100000	0-10	Pulse	1 x per day	1
11	Interactive effects of maternal provision and direct feeding	Maternal food quality and larval food densities	*Dunaliella tertiolecta, Chaetoceros muelleri*	0, 1000, 10000	ng	Pulse	ng	ng
12	Measure selectivity and food uptake	Different algae	*Phaeodactylum tricornutum, Pavlova lutheri, Tisochrysis lutea, Dunaliella sp. and Chaetoceros sp.*	1000 and 2500	ng	Only short-term experiments	Once	0.6-1.5
13	Size, development, settlement and mortality	Algae concentrations	*Proteomonas sulcata*	1000-50000	0-5	Pulse	1 x day	1
14	Development, survival and settlement assays	3 Algal densities	*Proteomonas sulcata*	1000-100000	0.1-10	Pulse	1 x per day	First days 2.5, subsequent: 1.2

Supplementary Table 2 Extended version of the larval density data (in larvae x ml^{-1}) for each of the experiments, treatments and nominal densities. For each point in time data represent averages (+- 1 SD, N = 5) over replicate larval cones, the density of each being estimated by 6 sub-replicate samples (not presented). Exception (2015 Experiment 4: only 3 replicate cones in high density treatment).

Experiment	Chlorophyll	Nominal density	Actual Density (larvae ml^{-1})					Mortality (M d^{-1})
		Date:	23/11/2015	25/11/2015	27/11/2015	30/11/2015	2/12/2015	
2015-2	H	0.5	0.46 (0.14)	0.53 (0.1)	0.41 (0.21)	0.38 (0.13)	0.31 (0.22)	0.07
	H	1	0.77 (0.24)	0.78 (0.07)	0.79 (0.13)	0.75 (0.21)	0.76 (0.17)	0.01
	H	2	1.65 (0.15)	1.61 (0.25)	1.66 (0.26)	1.39 (0.29)	1.17 (0.28)	0.05
		Date:	4/12/2015	7/12/2015	9/12/2015	11/12/2015	14/12/2015	
2015-3	L	0.5	0.56 (0.14)	0.55 (0.08)	0.47 (0.18)	0.52 (0.1)	0.43 (0.16)	0.03
	L	1	1.08 (0.21)	0.92 (0.16)	0.93 (0.17)	0.71 (0.14)	0.66 (0.15)	0.05
	L	2	2.02 (0.25)	1.97 (0.17)	1.71 (0.18)	1.7 (0.2)	1.49 (0.08)	0.04
		Date:	11/12/2015	14/12/2015	16/12/2015	18/12/2015	22/12/2015	
2015-4	H	0.5	0.53 (0.06)	0.56 (0.15)	0.55 (0.1)	0.57 (0.11)	0.41 (0.1)	0.04
	H	1	0.91 (0.15)	0.78 (0.1)	0.76 (0.15)	0.69 (0.28)	0.64 (0.14)	0.03
	H	2	1.94 (0.17)	1.85 (0.06)	1.87 (0.12)	1.83 (0.06)	1.69 (0.16)	0.01
2016		Date:	11/11/2016	14/11/2016	16/11/2016	18/11/2016	21/11/2016	
	H	0.5	0.6 (0.09)	0.65 (0.09)	0.58 (0.04)	0.71 (0.18)	0.53 (0.11)	0.02
	H	1	1.3 (0.16)	1.09 (0.28)	1.17 (0.1)	0.98 (0.25)	0.99 (0.25)	0.02
	H	2	2.29 (0.28)	2.26 (0.38)	2.21 (0.14)	2.23 (0.23)	2.14 (0.34)	0.01

L	0.5	0.6 (0.14)	0.69 (0.19)	0.67 (0.12)	0.65 (0.09)	0.51 (0.19)	0.04
L	1	1.18 (0.27)	1.17 (0.34)	1.26 (0.21)	0.96 (0.23)	1.01 (0.12)	0.03
L	2	2.38 (0.17)	2.12 (0.34)	2.16 (0.45)	2.23 (0.4)	1.93 (0.24)	0.01

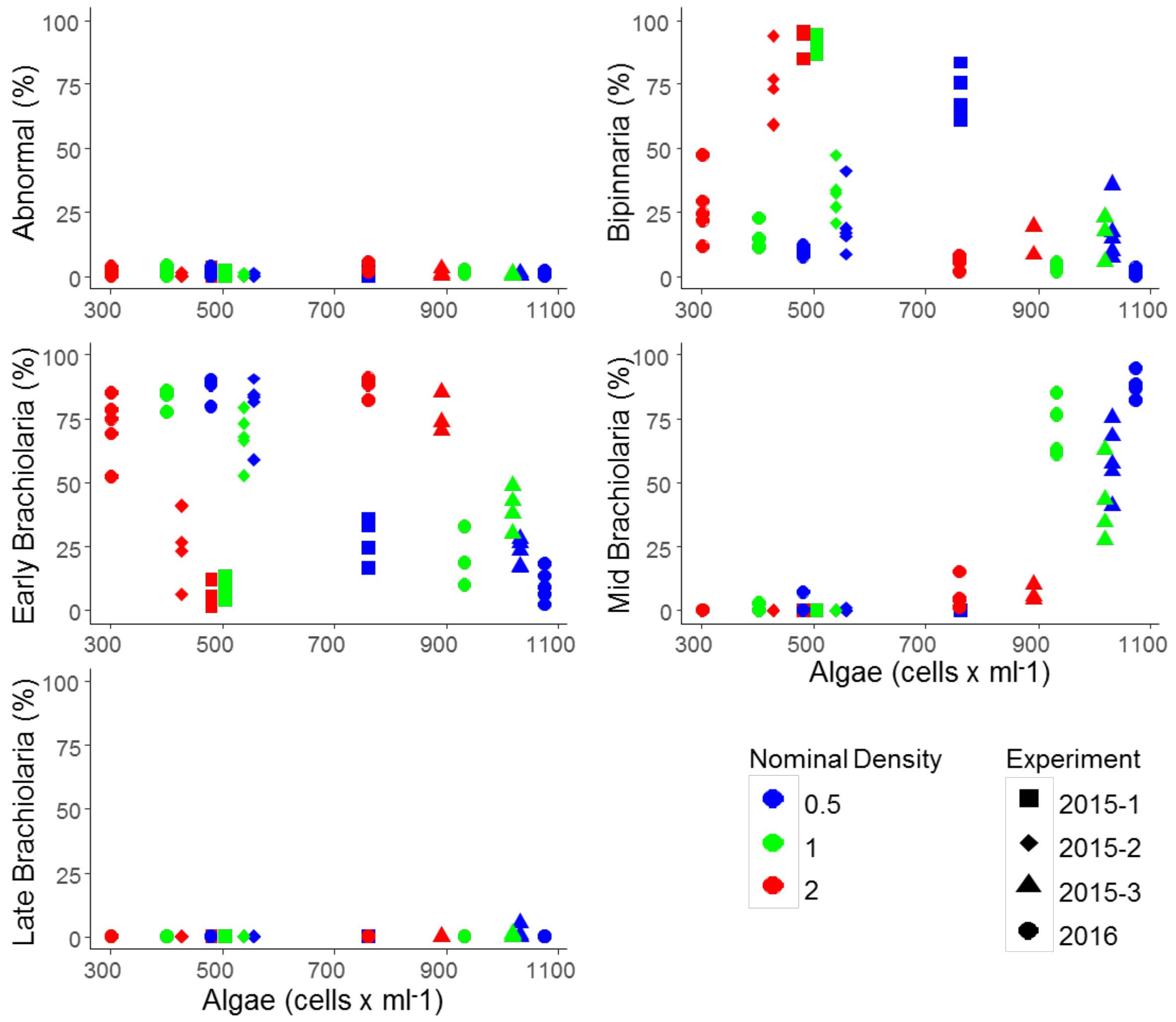

Supplementary Figure 1: The percentage of abnormally developed larvae (Abnormal), bipinnariae, early brachiolariae, mid-stage brachiolariae and late-stage brachiolariae of *Acanthaster cf. solaris* 10 days post fertilisation. Colours denominate the nominal density treatment, and the shape the experimental run (see legend).

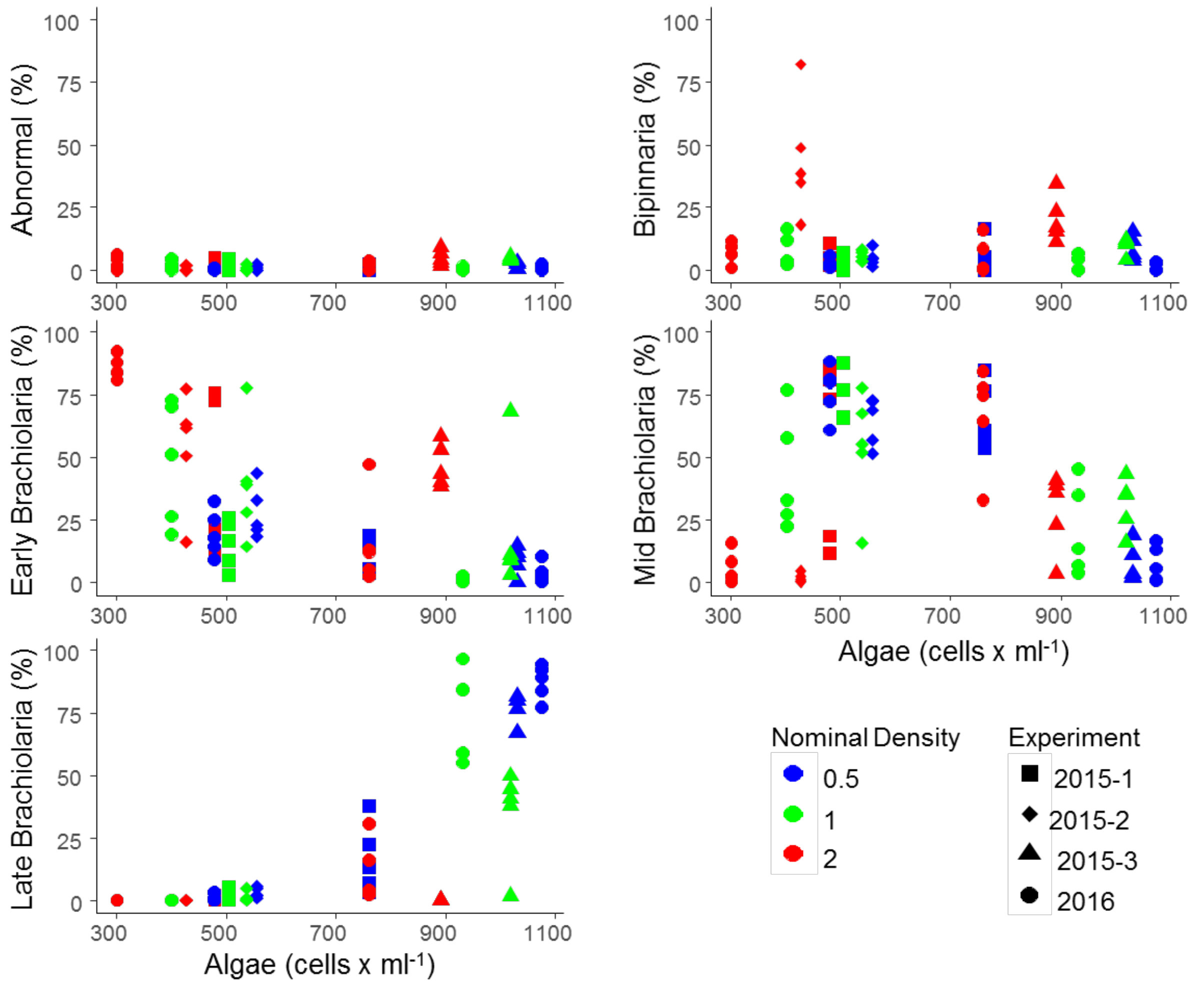

Supplementary Figure 2: The percentage of abnormally developed larvae (Abnormal), bipinnariae, early brachiolariae, mid-stage brachiolariae and late-stage brachiolariae of *Acanthaster cf. solaris* 15 days post fertilisation. Colours denominate the nominal density treatment, and the shape the experimental run (see legend).

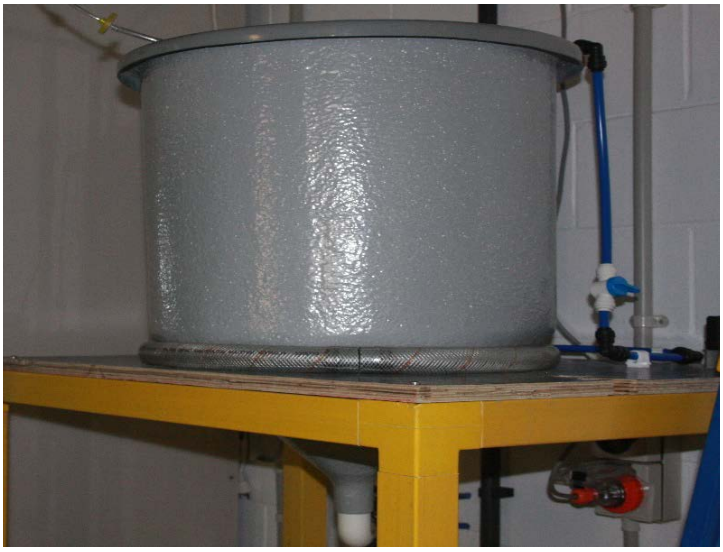

A

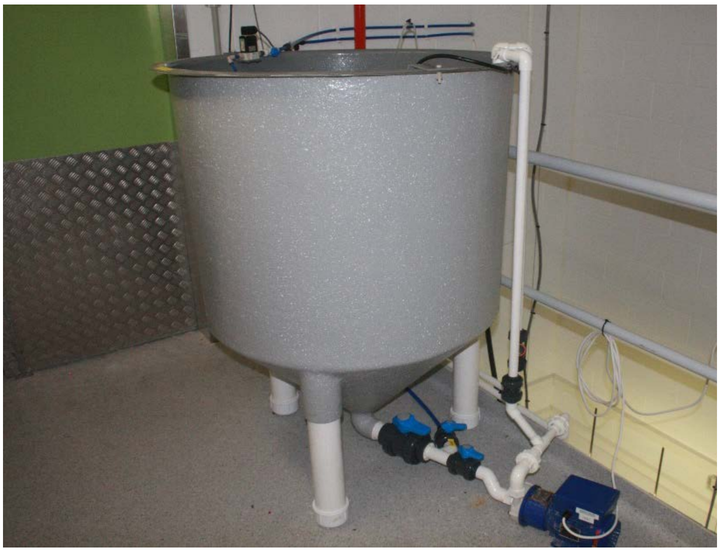

B

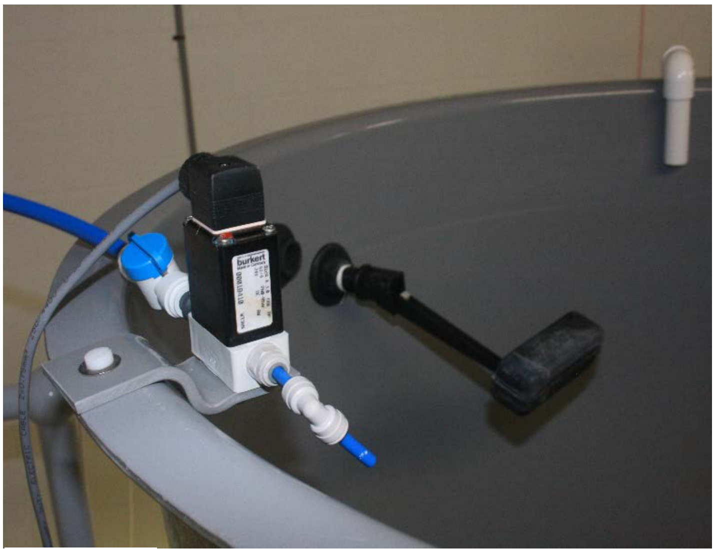

C

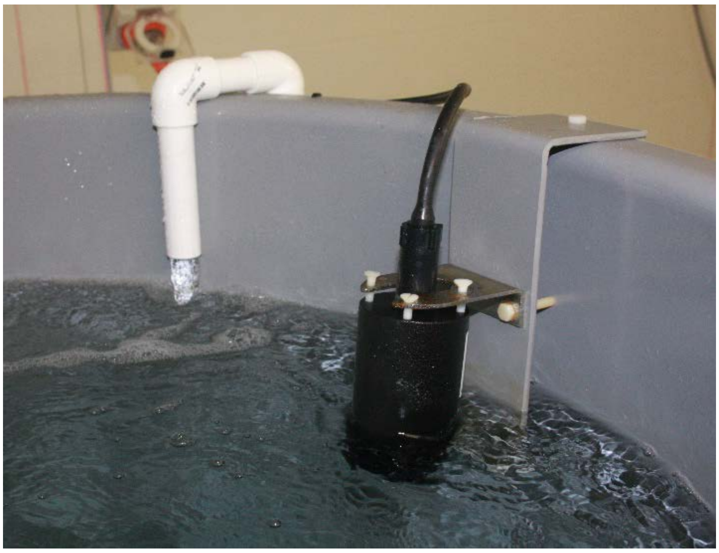

D

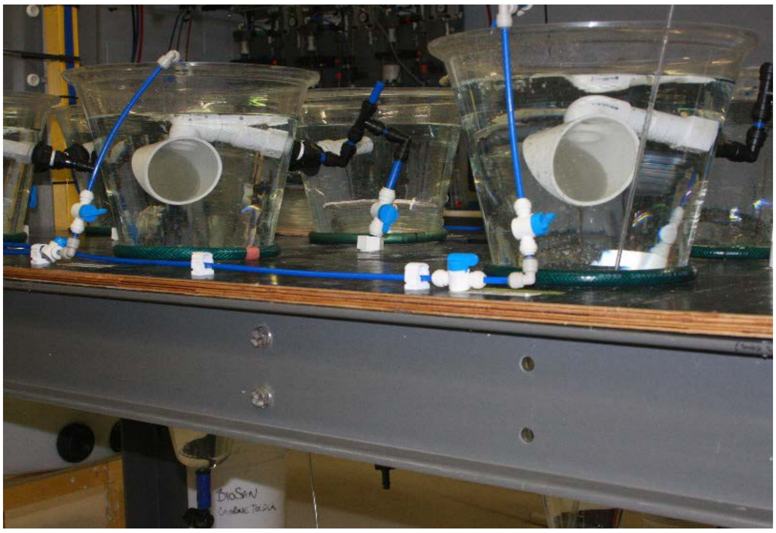

E

Supplementary Figure 3. Details of the automated feeding system. A) 70L Algae feeder tank, B) 500L header tank C) Solenoid valve with tank float, D) Fluorometer and E) 16L cones with Banjo filters supported in custom made benches.

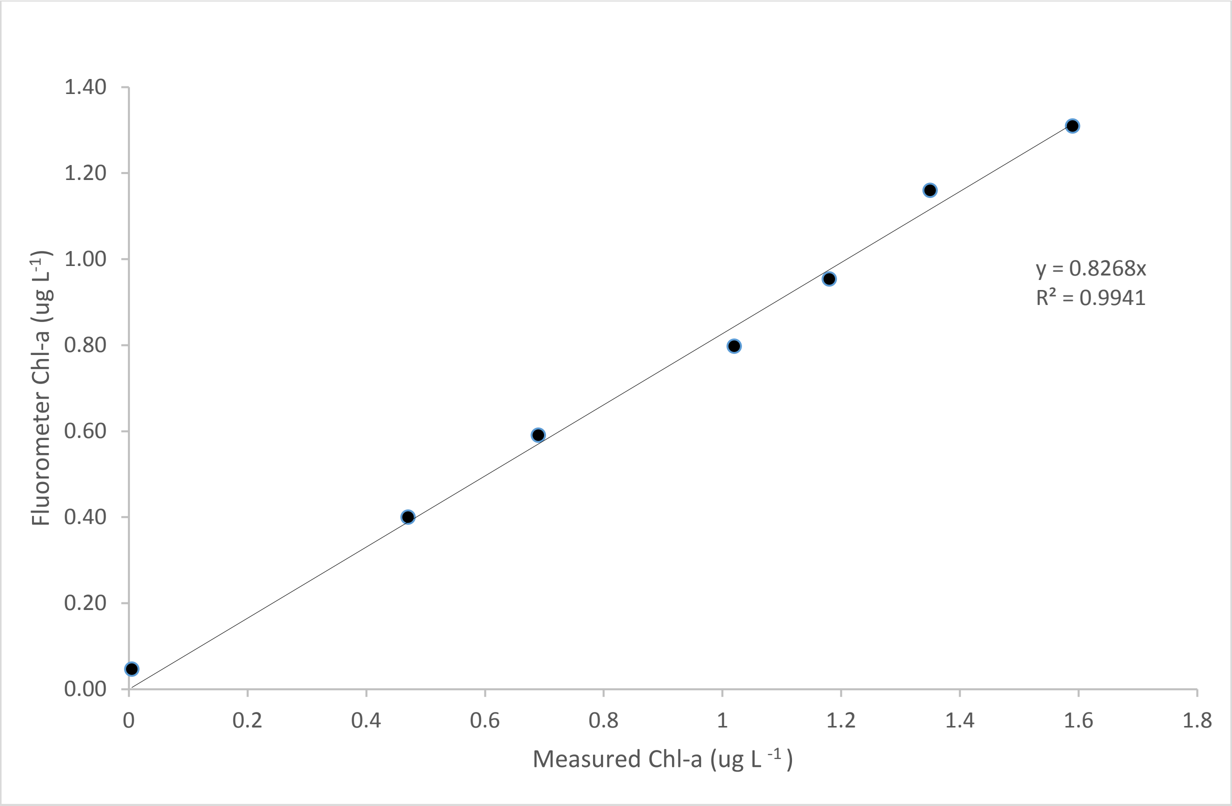

Supplementary Figure 4. Linear regression of chlorophyll a concentrations of measured and fluorometer determined values measured at the start of the 2016 experiment.

Supplementary References

1 Lucas, J. S. Reproductive and larval biology and *Acanthaster planci* (L.) in Great Barrier Reef. *Micronesica* **9**, 197-203 (1973).

2 Lucas, J. S. Quantitative studies on the feeding and nutrition during larval development of the coral reef asteroid *Acanthaster planci* (L.). *J. Exp. Mar. Biol. Ecol.* **65**, 173-193 (1982).

3 Ayukai, T. Ingestion of ultraplankton by the planktonic larvae of the crown-of-thorns starfish, *Acanthaster planci*. *Biological Bulletin, Marine Biological Laboratory, Woods Hole* **186**, 90-100 (1994).

4 Okaji, K. *Feeding ecology in the early life stages of the crown-of-thorns starfish, Acanthaster planci (L.)*, James Cook University, (1996).

5 Okaji, K., Ayukai, T. & Lucas, J. Selective feeding by larvae of the crown-of-thorns starfish, *Acanthaster planci* (L.). *Coral Reefs* **16**, 47-50 (1997).

6 Uthicke, S. *et al.* Impacts of ocean acidification on early life-history stages and settlement of the coral-eating sea star *Acanthaster planci*. *PLoS ONE* **8**, e82938, doi:10.1371/journal.pone.0082938 (2013).

7 Kamya, P. Z. *et al.* Larvae of the coral eating crown-of-thorns starfish, *Acanthaster planci* in a warmer-high CO_2 ocean. *Global Change Biology* **20**, 3365–3376, doi:10.1111/gcb.12530 (2014).

8 Uthicke, S. *et al.* Climate change as an unexpected co-factor promoting coral eating seastar (*Acanthaster planci*) outbreaks. *Scientific Reports* **5**, 8402, doi:10.1038/srep08402 (2015).

9 Wolfe, K., Graba-Landry, A., Dworjanyn, S. A. & Byrne, M. Larval starvation to satiation: Influence of nutrient regime on the success of *Acanthaster planci*. *PLoS ONE* **10**, e0122010 (2015).

10 Wolfe, K., Graba-Landry, A., Dworjanyn, S. A. & Byrne, M. Larval phenotypic plasticity in the boom-and-bust crown-of-thorns seastar, *Acanthaster planci*. *Marine Ecology Progress Series* **539**, 179-189 (2015).

11 Caballes, C., Pratchett, M. & Buck, A. Interactive effects of endogenous and exogenous nutrition on larval development for Crown-Of-Thorns Starfish. *Diversity* **9**, 15 (2017).

12 Mellin, C., Lugrin, C., Okaji, K., Francis, D. & Uthicke, S. Selective feeding and microalgal consumption rates by Crown-Of-Thorns Seastar (*Acanthaster cf. solaris*) larvae. *Diversity* **9**, 8 (2017).

13 Wolfe, K., Graba-Landry, A., Dworjanyn, S. A. & Byrne, M. Superstars: Assessing nutrient thresholds for enhanced larval success of *Acanthaster planci*, a review of the evidence. *Marine Pollution Bulletin* (2017).

14 Pratchett, M. S. *et al.* Larval survivorship and settlement of crown-of-thorns starfish (*Acanthaster cf. solaris*) at varying algal cell densities. *Diversity* **9**, 2 (2017).
